# A nomogram for predicting late radiation-induced xerostomia among locoregionally advanced nasopharyngeal carcinoma in intensity modulated radiation therapy era

**DOI:** 10.18632/aging.203308

**Published:** 2021-07-19

**Authors:** Kaixuan Yang, Wenji Xie, Xiangbin Zhang, Yu Wang, Arthur Shou, Qiang Wang, Jiangfang Tian, Jiangping Yang, Guangjun Li

**Affiliations:** 1 Department of Radiation Oncology, Cancer Center, West China Hospital, Sichuan University, Chengdu 610041, Sichuan, China; 2 Department of Radiation Oncology, West China Second University Hospital and Key Laboratory of Obstetrics and Gynecologic and Pediatric Diseases and Birth Defects of Ministry of Education, West China Second University Hospital, Sichuan University, Chengdu 610041, Sichuan, China; 3 West China School of Medicine, Sichuan University, Chengdu 610041, Sichuan, China; 4 School of Basic Medical Sciences and Forensic Medicine, Sichuan University, Chengdu 610041, Sichuan, China

**Keywords:** locoregionally advanced nasopharyngeal carcinoma, radiation-induced xerostomia, intensity modulated radiation therapy, volumetric modulated arc radiotherapy, platinum-based concurrent chemoradiotherapy

## Abstract

Background: Dry mouth sensation cannot be improved completely even though parotids are spared correctly. Our purpose is to develop a nomogram to predict the moderate-to-severe late radiation xerostomia for patients with locoregionally advanced nasopharyngeal carcinoma (LA-NPC) in intensity modulated radiation therapy (IMRT) / volumetric modulated arc radiotherapy (VMAT) era.

Methods: A dataset of 311 patients was retrospectively collected between January 2010 and February 2013. The binary logistic regression was to estimate each factor’s prognostic value for development of moderate-to-severe patient-reported xerostomia at least 2 years (Xer2y) after completion of radiotherapy. Therefore, we can develop a nomogram according to binary logistic regression coefficients. This novel model was validated by bootstrapping analyses.

Results: Contralateral Parotid mean dose (coMD<24.4Gy), VMAT (yes), and platinum-based concurrent chemoradiotherapy (no) were significantly related to patient-reported xerostomia at least 2 years (Xer2y) (all p < 0.001), and were included in the nomogram. Receiver operating characteristic (ROC) analysis revealed AUC (area under the ROC curve) with the value of 0.811 (0.710-0.912) of the nomogram, which was significantly higher than coMD 0.698 (0.560-0.840) from QUANTEC2010 (p<0.001). Calibration plots illustrated that the predicted Xer2y was close to the actual observation, and decision curve analyses (DCA) indicated valid positive net benefits.

Conclusion: We developed a feasible nomogram to predict patient-rated Xer2y based on comprehensive individual data in patients with LA-NPC in the real world. The proposed model is able to facilitate the development of treatment plan and quality of life improvement.

## INTRODUCTION

During the past decades, more than 80% of nasopharyngeal carcinoma (NPC) patients have prolonged survival after radical chemoradiotherapy. Concerns have arisen with late treatment-related side effects [1], such as xerostomia. It is the sensation of dryness resulting from salivary gland dysfunction or a variation in salivary structure. Decreased and/or thickened saliva is a common feature in xerostomia, which is usually related to oral health, speech, swallowing, and altered taste. Xerostomia with the Radiation Therapy Oncology Group (RTOG) grade 3/4 usually aggravates fatigue, sleeping domains and emotional functioning on quality of life (QoL) scales, which shows the diversified features of xerostomia [2, 3].

The QUANTEC (Quantitative Analyses of Normal Tissue Effects in the Clinic) Group proposed feasible protocols to prevent radiation-induced complications. For instance, severe xerostomia, defined as 25% of baseline in a stimulated salivary flow, could be relieved when at least one of parotids is spared with Dmean<20 Gy or both glands with Dmean<25 Gy [4]. However, the recommendation depends only on the dosimetric factors of the parotid glands. Even though parotids hypofunction contributes radiation-induced xerostomia, it is not the only prognostic factor [5]. Besides, the reduction in salivary function usually occurs in one week after the initiation of radiotherapy and continues thereafter. It takes approximately 2 years to recover after radiotherapy in most cases, which was confirmed by several patient self-reported questionnaires [6]. However, the majority of current studies focused on xerostomia less than 24 months [7, 8]. Actually, it is more reliable and reasonable to focus on 24-months monitor to avoid confounding from the gradual recovery of parotid function. Last but not least, methods used in salivary function measurements were unclear and fluctuated with 20–30% standard deviations for whole mouth evaluation [9, 10].

Recently, the patient-reported outcome has become the crucial step for normal tissue toxicity assessment and treatment tailoring. Compared with patient-reported events, xerostomia symptoms monitored by observer can underestimate the actual dryness symptoms [9, 11] Therefore, patient-reported outcomes should be reasonable research endpoints. It is essential to evaluate the patient-rated xerostomia two years after radiotherapy (Xer2y) based on large volumes of comprehensive clinical data.

Intensity modulated radiation therapy (IMRT), including IMRT with static ports and volumetric modulated arc radiotherapy (VMAT) IMRT, have been the most widely used forms of radiotherapy modalities for NPC. The previous planning studies have confirmed that VMAT improve parotid sparing without compromised target coverage compared to IMRT in terms of dosimetric parameters. However, it is unclear whether the improved parotid sparing can be translated into clinical benefits [12].

Therefore, it is meaningful to explore a general principle for preventing late xerostomia in contemporary technology. In this research, we developed and validated a novel nomogram to predict Xer2y based on a dataset of 311 individual data among patients with NPC in IMRT era.

## MATERIALS AND METHODS

### Patients

A dataset of 311 patients with stage III/IVa NPC (AJCC/UICC 8th edition) in our center from January 2010 to February 2013 was used in this research. We predefined the inclusion criteria as follows: 1) histologically detection confirmed by biopsy; 2) treatment with curative IMRT or VMAT, either alone or in combination with platinum-based chemotherapy; 3) without previous radiotherapy, surgery, and/or chemotherapy; 4) without previous malignancies; 5) without moderate-to-severe dry month before treatment, because we focus on radiation-induced xerostomia; 6) no acute (within 3 months after radiotherapy) xerostomia patients censored in 2 years are regarded as no Xer2y as a reason of parotid function recovery in most case [13]. The exclusion criteria were: 1) censored within 3 months after treatment; 2) patients with xerostomia censored within two years; This study has been authorized by the Research Ethics Board of our institution.

### Definition of dose-volume histograms (DVHs) and clinical factors

The following factors were used: tumor size, platinum-based dosage of chemotherapy, and DVH factors, et al (Table 1). The contralateral dose was defined as a larger proportion side of the parotid volume outside the PTV. Vd% was described as the parotid volume exposed in d Gy. Dv% was the minimal dose delivered into v% of the parotids volume. Treatment plans were restored, and DVHs parameters were extracted through our in-house script.

**Table 1:** Univariate and multivariate analyses for Xer2y.

	**Without xerostomia**	**With xerostomia**	**Univariate analysis**	**Multivariate analysis**		
	**(n = 233)**	**(n = 78)**	**P-value**	**OR**	**95%CI**	**p value**
Age(y)	47.4±10.9	47.6±9.6	0.845	1.145	0.834-1.57	0.403
19-30	10 (3.2)	4 (1.3)				
31-40	49 (15.8)	13 (4.2)				
41-50	87 (28)	31 (10)				
51-60	58 (18.6)	23 (7.4)				
61-74	28 (9)	7 (2.3)				
BMI	22 (20.4-24.2)	22.2 (20.2-24.5)	0.697			
Sex			0.838			
female/male	60 (19.3)/173 (55.7)	21 (6.8)/57 (18.3)				
Family history of cancer			0.314			
No/Yes	194(64.0)/39(12.5)	61 (19.6)/17 (5.5)				
Smoking			0.252			
No/Yes	122(39.2)/111(35.7)	35(11.3)/43(13.8)				
Alcohol			0.445			
No/Yes	166(53.4)/67(21.5)	52(16.7)/26(8.4)				
Histology			0.847			
Type1	1 (0.3)	0(0.3)				
Type2	215 (69.1)	74 (23.8)				
Type3	12 (3.9)	3 (10.0)				
Type4	5 (1.6)	1 (0.3)				
GTVnx(cm3)	53.0 (35.1-78.6)	55.5 (34.9-83.7)	0.448			
GTVnd(cm3)	15(8.5-29.6)	13.5 (8.4-24)	0.548			
T category			0.701			
T2/T3/T4	7(2.3)/133(42.8)/93(29.9)	1(0.3)/46(14.8)/31(10)				
N category			0.368			
N1/N2/N3	20(6.4)/136(43.7)/77(24.8)	4(1.3)/52(16.7)/22(7.1)				
8th stage			0.657			
III/ IVA	89(28.6)/144(46.3)	32(10.3)/46(14.8)				
PGTVnx (cGy)	6844(6668.1-6998)	6838(6514-7006.7)	0.449			
PGTVnd (cGy)	6815.2(6700.4-6924)	6762.6(6644.4-6800.4)	0.150			
VMAT			<0.001	0.031	0.004-0.236	**<0.001**
No/Yes	182(58.5)/51(16.4)	76(24.4)/2(0.6)				
IGRT			0.016	1.279	0.621-2.634	0.505
No/Yes	179(57.6)/54(17.4)	57(15.8)/21(9.3)				
RDD	47(45-50)	48(45-51)	0.107	1.029	0.991-1.068	0.135
Co						
MD (cGy)	3618(3345.2-3894.6)	3831(3440.7-4302.2)	<0.001	1.002	1.001-1.003	**<0.001**
V15 (%)	0.90(0.87-0.92)	0.90(0.85-0.92)	0.626			
V20 (%)	0.82(0.80-0.86)	0.83(0.81-0.85)	0.393			
V30 (%)	0.54(0.48-0.60)	0.53(0.47-0.61)	0.828			
V45 (%)	0.36(0.30-0.43)	0.37(0.31-0.43)	0.683			
D50	3612(3286-3874)	3570(3320-3777)	0.772			
Ip						
MD (cGy)	3700.4(3400-4085)	3631(3299-3906)	0.962			
V15 (%)	0.93(0.90-0.95)	0.92(0.88-0.95)	0.762			
V20 (%)	0.83(0.81-0.88)	0.85(0.81-0.88)	0.335			
V30 (%)	0.58(0.51-0.65)	0.56(0.48-0.67)	0.427			
V45 (%)	0.39(0.31-0.46)	0.38(0.32-0.44)	0.498			
D50	3780(3522-4181)	3831(3440-4387)	0.012			
Cetuximab			0.658			
No/Yes	199(64)/34(10.9)	65(20.9)/13(4.2)				
Chemotherapy						
IC			0.272	1.615	0.597-4.372	0.345
No/Yes	7(2.3)/71(22.8)	32(10.3)/201(64.6)				
CCRT			<0.001	4.60	2.20-9.596	**<0.001**
No/Yes	13(4.2)/65(20.9)	97(31.2)/136(43.7)				
AC			0.919	0.724	0.38-1.378	0.325
No/Yes	42(13.5)/36(11.6)	127(40.8)/106(34.1)				
IC-CCD	140(67-186.3)	142.6(66-204.6)	0.156			
CCRT-CCD	75.4(0-151.3)	90.6(68.4-179.3)	0.011			
AC-CCD	0(0-114.7)	0(0-143.2)	0.166			
IC*CCRT*AC			0.92			

RDD, radiotherapy duration days; OR, odds ratio; Co, Contralateral Parotid; 95% CI, 95% confidence interval; IP, Ipsilateral Parotid; MD, mean dose; CCD, cumulative cisplatin dose. Type1, Keratinizing SqCC, Type2, Non-Keratinizing Differentiated, Type3, Non-Keratinizing, Undifferentiated, Type4, Other/Unspecified.

### Treatment

Gross tumor volume of nasopharynx (GTVnx) and gross tumor volume of cervical lymph node (GTVnd) were defined as visible tumour and the positive lymph nodes, respectively. Clinical target volume (CTV)-1 contained nasopharynx primary tumour with an additional a 5–10mm margin (2–3mm posteriorly adjacent to the spinal cord or brainstem). CTV-2 contained CTV-1 with the selective neck IB to V area and subclinical sites. GTVnx/nd, CTV-1 and CTV-2 are prescribed to 69.96/73.92, 59.4, and 54 Gy, respectively, with 33-fraction (2.12 or 2.24 Gy per fraction) scheme for 6-7 weeks using 6-MV photons. Over 95% of the prescribed doses are acceptable. IMRT in this research included less than 9 fixed-field beam angles and adopted the step-and-shoot technique, whereas VMAT included a large number of beam directions from the arc trajectory and supports the simultaneous variation in gantry rotation and dose delivery (Supplementary Materials). The regimens of induction chemotherapy contained TPF (docetaxel 60 mg/m^2^ IV on day 1, cisplatin, 75 mg/m^2^ IV on day 1 or within 3 days, 5-FU 600 mg/m^2^ IV on days 1 to 5), which was repeated every 3 weeks for 2–3 cycles. Concurrent chemoradiotherapy consisted of each of 2 regimes: Cisplatin 80 mg/m2 IV every 3 weeks; Cisplatin 30–40 mg/m2 IV weekly. For patients who received adjuvant chemotherapy, PF (cisplatin, 80 mg/m2 IV on day 1, 5-Fu 800 mg/m2/d continuously IV on day 1–5) or TPF regimen was repeated every 3 weeks for 1–4 cycles. Chemotherapy dose can be adjusted according to hematological or non-hematological toxicity.

### Endpoints

As was shown in the European Organization for Research and Treatment of Cancer (EORTC) head and neck cancer module (QLQ-H&N35) questionnaire (Supplementary Materials), dry mouth item and sticky saliva item were utilized to quantify patient self-reported xerostomia in a 4-point Likert scale (i.e. none, a bit, quite a bit, to a lot). Each item score represents the degree of xerostomia. We defined moderate-to-severe patient-reported xerostomia at least 2 years (Xer2y) after completion of radiotherapy as the endpoint [7]. It corresponded with “quite a bit” to “a lot” on the 4-point scale.

### Statistical analysis

To illustrate the proposed predictive nomogram to models the relationship between a set of predictors and a binary xerostomia response variable, we first conducted an univariate logistic regression analysis to evaluate the Xer2y-prediction ability of each factor and interaction of chemotherapy. For those factors with p < 0.15 in the previous step, we then assessed them in stepwise binary logistic regression which was adjusted by age, Induction Chemotherapy (IC) and Adjuvant Chemotherapy (AC). OR were calculated with the logistic regression mode, Finally, we incorporated significant factors into nomogram according to binary logistic regression using the rms package in R. In particular, the Spearman rank correlation analysis was applied before the stepwise binary logistic regression to decrease the degree of multicollinearity.

The proposed model was validated internally by 1000 bootstrap resamples. We utilized the receiver operating characteristic (ROC) analysis to compare the sensitivity and specificity, and to find the optimal cutoff value. In addition, we used the calibration curve to compare the actual Xer2y against the prediction probability. Decision curve analysis (DCA) illustrated the clinical use of our model by calculating net benefits of the continuous threshold probabilities, which is iterated by putting into the true positives and waving the false positives.

All computations were conducted using SPSS (IBM 22.0) and R software (version 3.5.2). P < 0.05 was recognized as statistically significant.

## RESULTS

### Patient characteristics

**Figure 1:**
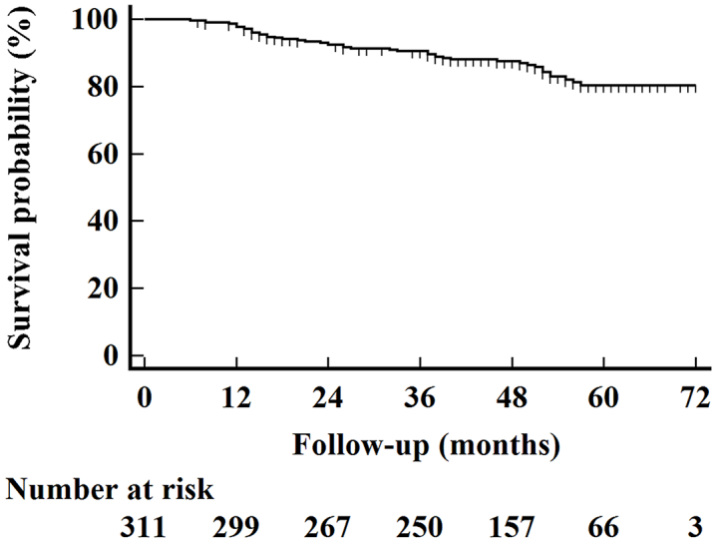
Kaplan–Meier overall survival curves for all 311 patients.

We summarized patient characteristics in Table 2. In general, 78 (25.1%) patients were diagnosed with Xer2y. As shown in Figure 1, The median OS time for the entire cohort was 49 months, and the 5-year OS rate was 80.3% (95% CI 77.4%-83.2%) with a median follow-up of 49 months (ranging from 6 to 74 months).

**Table 2:** Patient characteristics (n = 311).

**Characteristics**	**Patients n=311** **median (range)/** **No. of patients (%)**
Age(years), Mean (SD)	47.5 (36.9-58.1)
BMI, Median (IQR)	22 (20.4-24.5)
Gender	
Female/male	81 (26)/230 (74)
Family history of cancer	
No/yes	255 (82.0)/56 (18.8)
Cigarette smoking	
No/Yes	157 (50.5)/154 (49.5)
Alcohol	
No/Yes	218 (70.1)/93 (30.0)
Histology	
Keratinizing SqCC	1 (0.3)
Non-Keratinizing, Differentiated	289 (92.9)
Non-Keratinizing, Undifferentiated	15 (4.8)
Other/Unspecified	6 (1.9)
GTVnx(cm3), Median (IQR)	55.2 (35.1-78)
GTVnd(cm3), Median (IQR)	15 (8.5-28.5)
T category	
T2/T3/T4	8 (2.6)/179 (57.6)/124 (39.9)
N category	
N1/N2/N3	24 (7.7)/188 (60.5)/99 (31.8)
8th UICC/AJCC stage	
III/ IVA	121 (38.9)/190 (61.1)
PGTVnx (cGy), Median (IQR)	6842.4 (6631.7-6999)
PGTVnd (cGy), Median (IQR)	6807 (6694-6900)
IMRT/VMAT	258 (83)/53 (17)
IGRT	
No/Yes	236 (75.9)/75 (24.1)
Radiotherapy duration days	48 (45-50)
Cetuximab	
No/Yes	264 (84.9)/47 (15.1)
Chemoradiotherapy	
IC/CCRT/AC	272 (87.5)/201 (64.6)/142 (45.7)

BMI, body mass index; IQR, interquartile range; SD, standard deviation; IC, Induction Chemotherapy; CCRT, Concurrent Chemoradiation Radiotherapy; AC, Adjuvant Chemotherapy; IGRT, image-guided radiotherapy; PGTV, planning gross target volume; VMAT, volumetric modulated arc radiotherapy.

### Univariate and multivariate logistic regression analyses

In univariate logistic regression analysis, VMAT, image-guided radiotherapy (IGRT), radiotherapy duration days (RDD), coMD, ipsilateral Parotid D50 (ipD50), platinum-based concurrent chemoradiotherapy (CCRT), and CCRT cumulative cisplatin dose (CCD) were statistically associated with Xer2y in Table 1 (p < 0.15). According to Spearman’s analyses, the significant collinearity between CCRT and CCRT-CCD (r = 0.781, p < 0.001). coMD and ipD50 (r = 0.96, p < 0.001) were revealed in Table 3. We included CCRT and coMD instead of CCRT-CCD and ipD50 in the binary logistic regression by the use of a published selection criteria [14]. Age, IC and AC were also considered as adjusted factors. Therefore, age, IC, AC, VMAT, IGRT, RDD, coMD and CCRT were incorporated into the binary logistic regression: coMD (OR: 1.002, 95%CI: 1.001-1.003, p <0.001), VMAT (OR:0.031, 95%CI: 0.004-0.236, p <0.001), and CCRT (OR:4.60, 95%CI: 2.20-9.596, p <0.001) were predictors of Xer2y (Table 1). Since the majority of eligible patients did not receive cetuximab, for 264 patients who had not received the target therapy, 65 (25%) experienced Xer2y. After a subgroup binary logistic regression in these specific patients, as was noted previously, coMD (OR: 1.002, 95%CI: 1.001-1.003, p <0.001), VMAT (OR: 0.031, 95%CI: 0.004-0.242, p =0.001), and CCRT (OR: 5.389,95%CI: 2.375-12.23, p <0.001) were still significantly related with Xer2y.

**Table 3:** Critical values (p < 0.15 in univariate test) for Spearman’s rank correlation analyses.

	**VMAT**	**IGRT**	**RDD**	**coMD**	**ipD50**	**CCRT**	**CC-CCD**
VMAT	1	0.052	0.074	0.046	-0.039	0.049	0.101
IGRT	0.052	1	0.002	0.069	0.054	-0.012	-0.055
RDD	0.074	0.002	1	-0.049	-0.075	-0.018	-0.003
coMD	0.046	0.069	-0.049	1	0.96***	0.094	0.044
ipD50	-0.039	0.054	-0.075	0.96***	1	0.084	0.042
CCRT	0.049	-0.012	-0.018	0.094	0.084	1	0.781***
CC-CCD	0.101	-0.055	-0.003	0.044	0.042	0.781***	1

*, P < 0.05; **, P < 0.01; ***, P < 0.001.

### Nomogram development and validation

**Figure 2:**
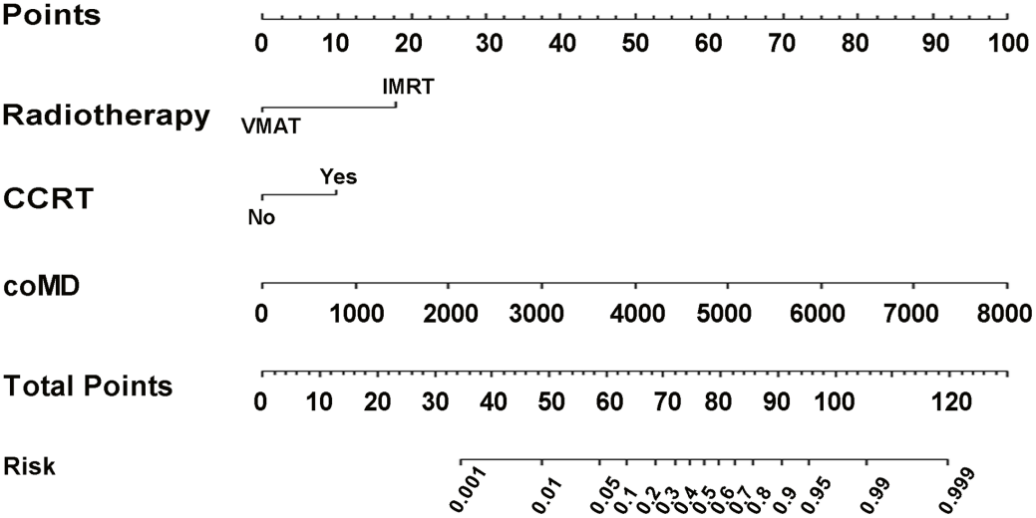
**Nomogram of Xer2y occurrence prediction.** For each individual patient, the value of three variables (VMAT, CCRT and coMD) are translated into points by projecting them into the upper-most line (point scale), respectively. Summing the points of the three variables and projecting the total points value downward to the bottom-most line can determine the probability of this patient to have Xer2y (patient-reported xerostomia at least 2 years after radiotherapy). VMAT (volumetric modulated arc radiotherapy). CCRT (platinum-based concurrent chemoradiotherapy). coMD (Contralateral Parotid mean dose).

**Figure 3:**
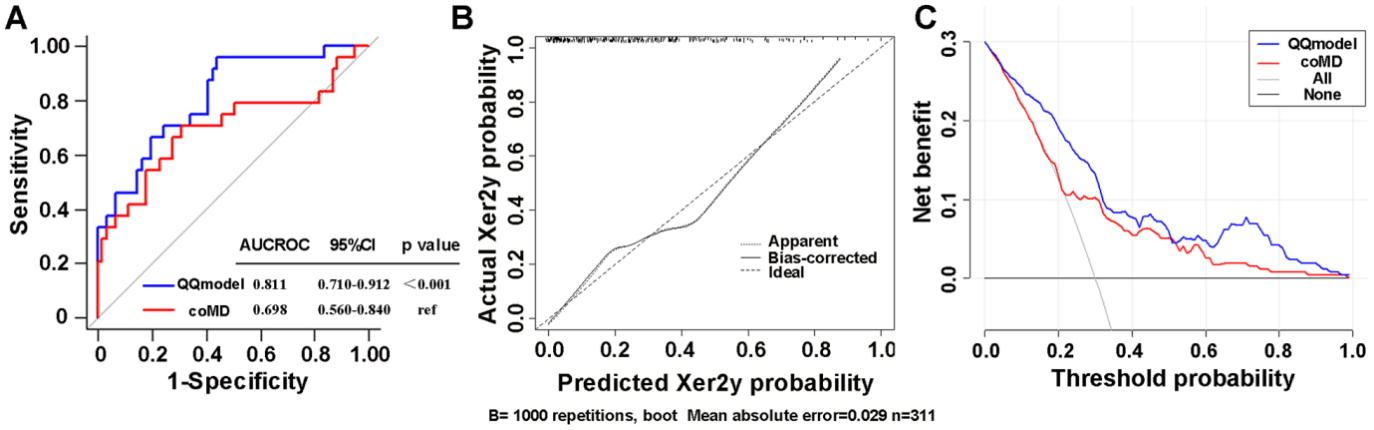
(**A**) ROC curves comparing the sensitivity and specificity of the coMD and nomogram (**B**) Calibration plots of the nomogram for Xer2y incidence prediction. X-axis indicates the predicted probabilities with Xer2y while y-axis shows the actual events. The ideal prediction will correlate when slope equals to 1 (the black broken line in the figure). (**C**) Decision curves of two risk models for Xer2y incident prediction. The horizontal axis represents the risk threshold while the vertical-axis denotes standardized net benefit. The solid black line is the net benefit when no patients have Xer2y while the dash gray line suggests the net benefits where patients have Xer2y at a certain risk threshold. The red and blue curves imply the results of the Xer2y on the basis of coMD and nomogram, respectively. AUCROC (area under the receiver operating characteristic curve). 95% CI (95% confidence interval).

According to coefficients of the multivariate logistic regression analyses, a nomogram (namely QQmodel) was developed visually to predict Xer2y (Figure 2). The QQmodel was internally validated using the bootstrapping analyses. As the ROC curves shown in Figure 3A, the QQmodel gained an AUC of 0.811 (95%CI: 0.71–0.912), which was significantly superior (p<0.001) to coMD (0.698, 95%CI: 0.560–0.840). The cut-off value (dose–volume constraints) of coMD was 24.4 Gy. The proposed nomogram also showed promising prediction efficiency (sensitivity: 56%, specificity: 96%). Calibration plots of QQmodel performed well as the predicted Xer2y and the actual observation gained an agreeable consistency (Figure 3B). DCA illustrated that QQmodel achieved much more positive net benefits than coMD for the majority of the threshold probabilities, which demonstrated an encouraging clinical effect of the QQmodel (Figure 3C).

## DISCUSSION

It is widely acknowledged that dysfunction of the parotids is one of the most problematic late effects in head and neck cancer patients [5]. We established and validated the first nomogram to predict the occurrence of moderate-to-severe Xer2y in IMRT/VMAT era. Results of the present study indicated that coMD, VMAT and CCRT are independent prognosticators of moderate-to-severe Xer2y from comprehensive clinical and dosimetric factors among patients with LANPC. We proved our model is superior to coMD from QUANTEC2010 [4] criteria.

The predictive ability of coMD (<24.4 Gy) for Xer2y was confirmed in our model. After QUANTEC2010 published, Recent research found that the contralateral mean parotid gland dose and baseline xerostomia are the top two important predictors to patients-reported QoL for dry mouth and 51.6% cases studies suffered from moderate-to-severe Xer6m in that study [15, 16]. Given that some xerostomia will steadily restore in around 2 years, 25.1% of Xer2y in the present research will be biologically plausible. Parotid glands mean dose is a significant risk factor for patient-reported moderate-to-severe xerostomia for post treatment within 6, 12, 18 and 24 months [7]. Furthermore, xerostomia was closely related to parotid glands mean dose in the actual delivered dose research [5, 17]. These evidences suggest that coMD could play a crucial role in radiation xerostomia.

In the present study, the dosimetric parameters of parotid gland V15, V20, V30, V45 and D50 were not significant predictors of Xer2y. There are several existing explanations. Firstly, V15, V30, V45 were assessed by observer-based grading [18]. Relationship between patient-reported events and observer-based grading have been verified to be inconsistent [9, 11]. Secondly, V20 is from the delivery dose rather than planning dose [17].

Compared with IMRT, we found that VMAT was better at preventing Xer2y. This finding supported the previous work [19]. Furthermore, we revealed that VMAT conserves salivary function when coMD is established. A possible explanation is that other salivary glands, such as the oral cavity accessory glands also play a key role in the sensation of chronic dry mouth apart from the parotid gland [20, 21]. Since IMRT is characterized by typically arranging less than 9 fixed-field beam angles, VMAT instead has numerous beam directions from the arc trajectory. Thanks to there being no beam modulation by the MLCs, VMAT achieves less high-dose areas, more homogeneous dose distribution, and better spare to the salivary glands while treatment beams are sequenced one after another in IMRT treatment plan [22]. Some researchers found that the isodose distribution in IMRT approaches to VMAT as increasing numbers of beams are utilized [23]. Moreover, VMAT has more beam entry angles and supports the simultaneous variation in MLC leaf positions, gantry rotation speed and dose rate, resulting in great reductions in monitor units and treatment delivery time per fraction by transforming IMRT into VMAT for head and neck cancer [24]. Lower MU has its advantages such as the potential decrease in total body dose due to leakage and scattering from MLCs [25, 26]. Extra low-dose areas may have potential protective effects on other oral cavity accessory glands [12]. Reducing treatment time increases patient comfort and decreases intra-fractional movement [27]. However, other studies mentioned that VMAT is not superior to IMRT in terms of dry month [28]. This inconsistency probably results from planning objectives, dose calculation algorithm and patient characteristics. Therefore, conditions should be declared prior to the clinical use.

Our research implied that CCRT, which was more powerful than CCRT-CCD, was an independent prognosticator for moderate-to-severe Xer2y. This conclusion confirmed several previous works [29]. Nonetheless, other published studies failed to reveal increased xerostomia in CCRT [30], because their datasets were moderate-to-severe xerostomia patients within 2 years after radiotherapy and gradual recovery of parotid glands function might attribute to unmeasured confounding. Recently, alternative concurrent chemotherapy regimens with mild toxicity for patients with LA-NPC have been reported [31]. According to our knowledge, there is no published data from randomized control trial to address the role of CCRT with IMRT versus IMRT alone for LA-NPC.

Validation is required to highlight the contribution of the proposed nomogram. External validation is usually a gold standard method. However, because of the modest sample size, even researchers in some eminent institutes preferred carrying out an internal validation. As one of the internal validation, the bootstrapping analysis, such as the calibration curve, is advantageous especially for a relatively small sample size [32]. However, conditions should be declared before the clinical use of model.

This work used the patient self-reported moderate-to-severe xerostomia at least 2 years (Xer2y) after completion of radiotherapy quantified by the EORTC QLQ-H&N35 questionnaire as the endpoints. Quality of life (QoL) reflects the awareness of the consequences of the disease and the burden that the disease imposes on the individual's daily function. This is performed by patients’ own sense. EORTC QLQ-H&N35 was one of the most widely used tools to reveal the shift of QoL [6]. As one of significant problems in QoL, Xerostomia (common feature of decreased and/or thickened saliva) has been successfully quantified by the Xerostomia item in EORTC QLQ-H&N35 [7]. Compared with xerostomia symptoms assessed by physicians, patient self-reported events were proved to be more reasonable [9]. However, other items associated with xerostomia-related QoL, such as global health status, swallowing, social eating and social contact, should be further collected and investigated in the future.

The AUC of coMD was 0.698(0.560-0.840), which was similar to former dosimetric study [15]. Such common predictive ability elucidates that salivary flow preservation due to accurately spared parotids fails to improve dry mouth sensation or of quality of life effectively [33]. In this research, the AUC of integration nomogram of coMD, VMAT and CCRT was increased to 0.811(0.710-0.912). Although parotids and submandibular glands are proposed to be spared in QUANTEC2010 and the Radiation Therapy Oncology Group 1016 trial in 2011, only 90% of bilateral parotids were outlined and submandibular glands were not delineated in 56% out of treatment plans in public hospitals [34], not to say other salivary glands. Therefore, our results have great significance for clinical work.

Some limitations of the present study are addressed here. Firstly, the research did not take position error and anatomical variation into consideration. Given the retrospective nature of this study design. our Cone-beam Computed Tomography data was insufficient for analysis. Further study should be conducted to assess their role in the nomogram. Secondly, the endpoint in this research was late xerostomia 2 years after radiotherapy. Although it helped to avoid interference with parotid function recovery, it might result in selective bias from unmeasured and unknown confounders as a reason of the inclusion / exclusion criteria. Thirdly, our participant samples were in a modest size and were taken from an endemic area in China. Fourthly, it is difficult to completely avoid effort and alertness of bias in planning optimization, although clinical plans with the same goals [35] are validated by a senior Medical Dosimetrist in our department. Finally, the reality of the nomogram would be more persuasive if an external validation is executed.

In conclusion, we established a clinically feasible nomogram involving coMD (<24.4 Gy), VMAT (yes), and CCRT (no) that could quantify the risk for patient-rated Xer2y among LA-NPC based on large volumes of comprehensive clinical data in IMRT/VMAT era. This novel predictive model reduced intervention from parotid function recovery, and will be valuable for the development of treatment plan and the improvement of quality of life.

## Supplementary Material

Supplementary Materials

Supplementary EORTC QLQ-H&N35 questionnaire
