# Supplementary material for: A nomogram for predicting late radiation-induced xerostomia among locoregionally advanced nasopharyngeal carcinoma in intensity modulated radiation therapy era
Source: Aging (Albany NY). 2021 Jul 19;13(14):18645–57. doi: 10.18632/aging.203308 (PMC8351700; doi:10.18632/aging.203308)
Supplement: Supplementary Materials [file aging-13-203308-s001.pdf]

SUPPLEMENTARY MATERIAL

Planning techniques

VMAT plan

The Elekta Synergy linear accelerator (Elekta, Stockholm, Sweden) was utilized to perform IMRT/VMAT. We adopted an adjusted dose rate during the execution of VMAT. When the system was implemented and tested, it showed that the most stable dose rate for 6-MV photon beams was 530 MU/min, which then in this study was set as the first level in the dose rate table, followed by 265 MU/min, 132 MU/min, 66 MU/min, 33 MU/min, 16 MU/min, and 8 MU/min. The table served as a reference of dose rates during treatment planning.

We adopted the Philips Pinnacle^3 Planning System v.9.0 with SmartArc module for VMAT planning. For the dose calculations with the collapsed cone convolution-superposition method and a dose grid resolution of 3 mm, the heterogeneity option was turned on. The system was set to create two full arcs, which in practice were a rotation of 358°, from 181° to 179° clockwise (CW) and counterclockwise (CCW) gantry rotation. Angular control point (CP) spacing was set at 4°, and a total of 91 control points were created for each arc.

Conventional IMRT

We used the Philips Pinnacle^3 Planning System v.9.0 for IMRT planning. Direct machine parameter optimization (DMPO) module was adopted for the planning, which used 7–9 angles to evenly separate coplanar fields. The minimum segment area was set to 5 cm^2, and minimum segment MU was 4-5 MUs. A collapsed-cone convolution-superposition algorithm was used to calculate dosage, with a dose grid resolution of 3 mm.
