# Supplementary material for: A nomogram for predicting late radiation-induced xerostomia among locoregionally advanced nasopharyngeal carcinoma in intensity modulated radiation therapy era
Source: Aging (Albany NY). 2021 Jul 19;13(14):18645–57. doi: 10.18632/aging.203308 (PMC8351700; doi:10.18632/aging.203308)
Supplement: Supplementary EORTC QLQ-H&N35 questionnaire [file aging-13-203308-s002.docx]

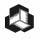


**EORTC QLQ - H&N35**

Patients sometimes report that they have the following symptoms or problems. Please indicate the extent to which you have experienced these symptoms or problems during the past week. Please answer by circling the number that best applies to you.

| **During the past week:** | | **Not** | **A** | **Quite** | **Very** |
| --- | --- | --- | --- | --- | --- |
|  |  | **at all** | **little** | **a bit** | **much** |
| 31. | Have you had pain in your mouth? | 1 | 2 | 3 | 4 |
| 32. | Have you had pain in your jaw? | 1 | 2 | 3 | 4 |
| 33. | Have you had soreness in your mouth? | 1 | 2 | 3 | 4 |
| 34. | Have you had a painful throat? | 1 | 2 | 3 | 4 |
| 35. | Have you had problems swallowing liquids? | 1 | 2 | 3 | 4 |
| 36. | Have you had problems swallowing pureed food? | 1 | 2 | 3 | 4 |
| 37. | Have you had problems swallowing solid food? | 1 | 2 | 3 | 4 |
| 38. | Have you choked when swallowing? | 1 | 2 | 3 | 4 |
| 39. | Have you had problems with your teeth? | 1 | 2 | 3 | 4 |
| 40. | Have you had problems opening your mouth wide? | 1 | 2 | 3 | 4 |
| 41. | Have you had a dry mouth? | 1 | 2 | 3 | 4 |
| 42. | Have you had sticky saliva? | 1 | 2 | 3 | 4 |
| 43. | Have you had problems with your sense of smell? | 1 | 2 | 3 | 4 |
| 44. | Have you had problems with your sense of taste? | 1 | 2 | 3 | 4 |
| 45. | Have you coughed? | 1 | 2 | 3 | 4 |
| 46. | Have you been hoarse? | 1 | 2 | 3 | 4 |
| 47. | Have you felt ill? | 1 | 2 | 3 | 4 |
| 48. | Has your appearance bothered you? | 1 | 2 | 3 | 4 |

Please go on to the next page

| **During the past week:** | | **Not** | **A** | **Quite** | **Very** |
| --- | --- | --- | --- | --- | --- |
|  |  | **at all** | **little** | **a bit** | **much** |
| 49. | Have you had trouble eating? | 1 | 2 | 3 | 4 |
| 50. | Have you had trouble eating in front of your family? | 1 | 2 | 3 | 4 |
| 51. | Have you had trouble eating in front of other people? | 1 | 2 | 3 | 4 |
| 52. | Have you had trouble enjoying your meals? | 1 | 2 | 3 | 4 |
| 53. | Have you had trouble talking to other people? | 1 | 2 | 3 | 4 |
| 54. | Have you had trouble talking on the telephone? | 1 | 2 | 3 | 4 |
| 55. | Have you had trouble having social contact with your family? | 1 | 2 | 3 | 4 |
| 56. | Have you had trouble having social contact with friends? | 1 | 2 | 3 | 4 |
| 57. | Have you had trouble going out in public? | 1 | 2 | 3 | 4 |
| 58. | Have you had trouble having physical |  |  |  |  |
|  | contact with family or friends? | 1 | 2 | 3 | 4 |
| 59. | Have you felt less interest in sex? | 1 | 2 | 3 | 4 |
| 60. | Have you felt less sexual enjoyment? | 1 | 2 | 3 | 4 |

| **During the past week:** | | **No** | **Yes** |
| --- | --- | --- | --- |
| 61. | Have you used pain-killers? | 1 | 2 |
| 62. | Have you taken any nutritional supplements (excluding vitamins)? | 1 | 2 |
| 63. | Have you used a feeding tube? | 1 | 2 |
| 64. | Have you lost weight? | 1 | 2 |
| 65. | Have you gained weight? | 1 | 2 |

© Copyright 1994 EORTC Qua lity of Life Study Group, version 1.0 All rights reserved
